# ﻿First record of the family Calliopiidae (Crustacea, Malacostraca, Amphipoda) from Korean waters, with description of new species *Calliopiusulleungensis* sp. nov.

**DOI:** 10.3897/zookeys.1221.139066

**Published:** 2024-12-20

**Authors:** Kyung-Won Kim, Young-Hyo Kim

**Affiliations:** 1 Department of Biological Sciences, Dankook University, Cheonan 31116, Republic of Korea Dankook University Cheonan Republic of Korea

**Keywords:** Amphipod, calliopiid, key, morphology, new record, taxonomy

## Abstract

A new species of the family Calliopiidae was collected from the East Sea of Korea. *Calliopiusulleungensis***sp. nov.** is similar to *C.columbianus* Bousfield & Hendrycks, 1997 in having numerous calceoli on the posteromedial margins of antennae and a weakly carinate body. However, the new species can be distinguished from *C.columbianus* by the shorter process on peduncular article 3 of antenna 1, subrectangular eyes, and fewer articles in the antenna flagellum. This species, along with *C.ezoensis*Shimoji et al. 2020, occurs in the Western Pacific. The females of the two species are morphologically very similar, but the males of *C.ezoensis* are easily distinguishable, as the gnathopod 1 is larger than gnathopod 2. The new species is fully illustrated and extensively compared with related species. In this paper, both *Calliopius* and Calliopiidae are reported from Korea for the first time. A key to species of *Calliopius* is also provided.

## ﻿Introduction

The family Calliopiidae G.O. Sars, 1893 represents a moderately sized group within the amphipods. Members of this family display a range of morphologically diverse forms that share many symplesiomorphies but only a few synapomorphies (Bousfield and Hendrycks 1997). In previous studies, the family was grouped with Eusiridae due to the following morphological similarities: well-developed eyes, body often with dorsal carina, slender antennae, generally degenerated accessory flagellum, feeble gnathopods, and uropod 3 with rami subsimilar in length (Sars 1893; Barnard and Karaman 1991; Bousfield and Hendrycks 1997). However, with the establishment of the suborder Senticaudata, the family Calliopiidae was reclassified and is now positioned in a category that places it systematically distant from Eusiridae (Lowry and Myers 2013).

The genus *Calliopius* Lilljeborg, 1865 currently comprises nine species (Horton et al. 2024). The genus was originally described as *Calliope* Spence Bate, 1857, with *Calliopeleachii* Spence Bate, 1857 as the type species. Later, as the genus *Calliopius* was established by Lilljeborg in 1865, *Calliope*leachii was considered a junior subjective synonym of *Amphithoelaeviuscula* Krøyer, 1838. The genus *Calliopius* is characterized by reduced accessory flagellum, fused on peduncular article 3; subchelate gnathopods, and entire telson (Bate 1857). In this paper, we describe and illustrate a new species of *Calliopius* in the Korean amphipod fauna. This is also first record of the family Calliopiidae from Korean waters. Additionally, we provide a key to *Calliopius* species.

## ﻿Materials and methods

**Figure 1.**
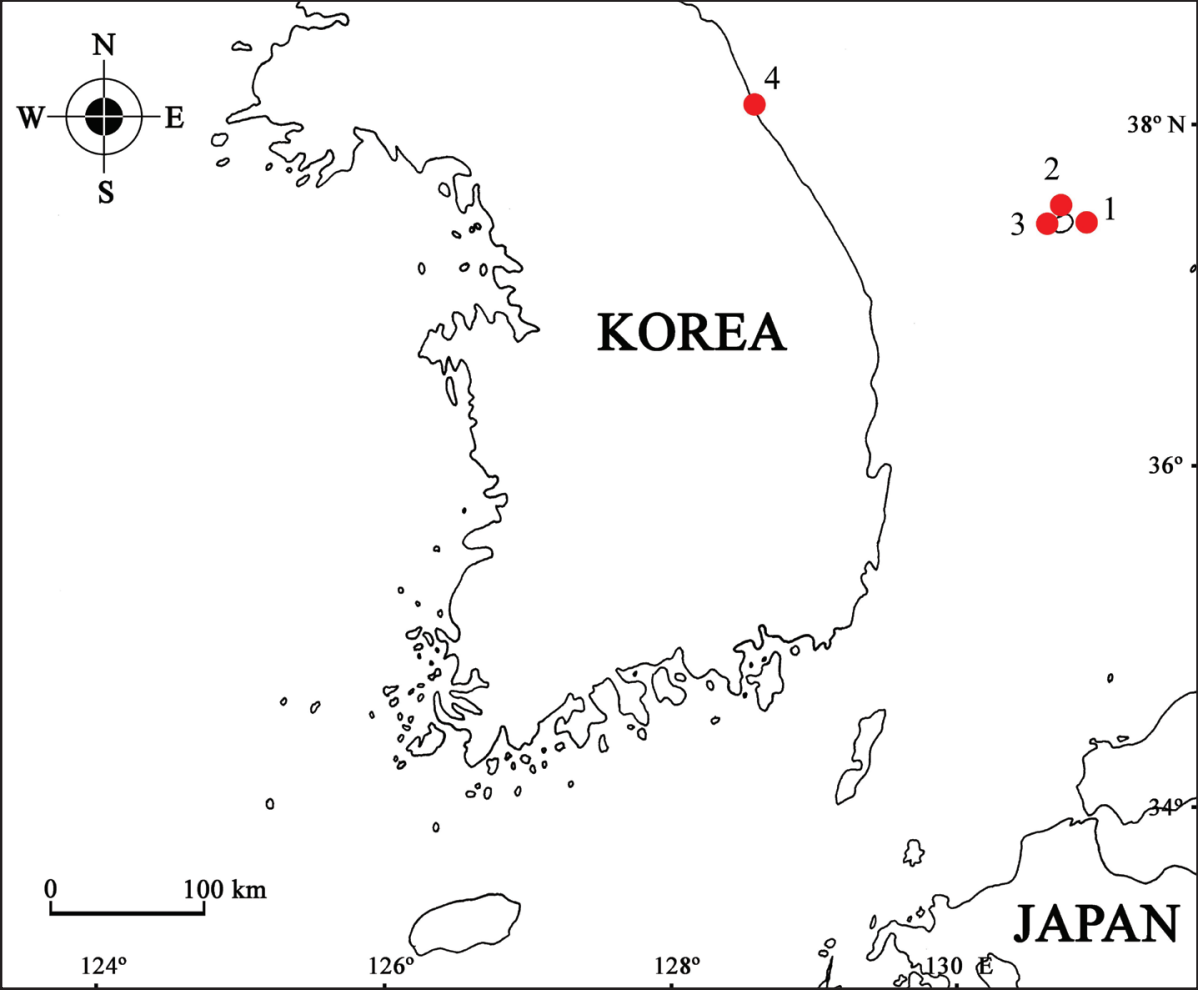
Collecting sites of the *Calliopiusulleungensis* sp. nov.: 1 = Ulleungdo Island (site 1); 2 = Ulleungdo Island (site 2); 3 = Taeha-ri; 4 = Bongpo-ri.

Specimens were collected from subtidal waters around Ulleungdo island, East Sea, Korea (Fig. 1). The collected specimens were fixed in 95% ethanol for preservation and later dissected in glycerol on Cobb’s aluminum hole slides. The materials were examined under stereoscopic (Olympus SZX 10) and compound microscopes (Olympus BX 51), and drawings and measurements were made with the aid of a drawing tube. Line drawings were produced using Clip Studio Paint software (Celsys, Japan). Body length was measured from the tip of the rostrum to the posterior end of the urosome, along the dorsal parabolic line of the body. The examined specimens are deposited at the National Marine Biodiversity Institute of Korea (**MABIK**), Seocheon, Korea, and the Department of Biological Science, Dankook University (**DKU**), Cheonan, Korea.

## ﻿Taxonomy

### ﻿Order Amphipoda Latreille, 1816

**Family Calliopiidae G.O. Sars, 1893**

**Korean name: Kal-li-o-pe-yeop-sae-u-gwa, new**

#### ﻿Genus Calliopius

Taxon classificationAnimaliaAmphipodaCalliopiidae

Lilljeborg, 1865

AFAEA6B8-1DFF-5C09-B479-0A60039D7316

##### Type species.

*Calliopiuslaeviusculus* (Krøyer, 1838).

#### ﻿ Calliopius ulleungensis sp. nov.

Taxon classificationAnimaliaAmphipodaCalliopiidae

0CBC3091-E512-5E9C-93BC-79039DA4C533

https://zoobank.org/85C8913F-106E-435B-9D09-456EEC991326

**Figure 2.**
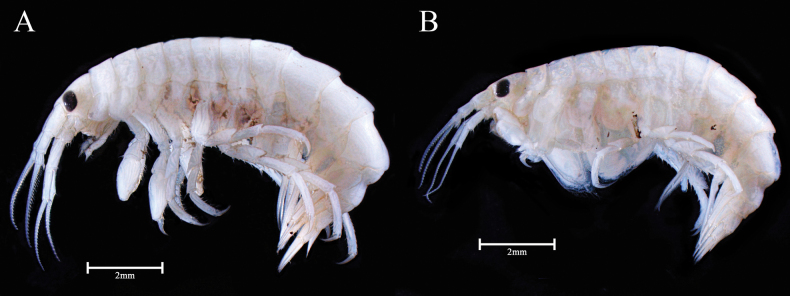
*Calliopiusulleungensis* sp. nov. **A** holotype male, 13.6 mm **B** paratype female, 11.6 mm. Scale bars: 2.0 mm.

**Figure 3.**
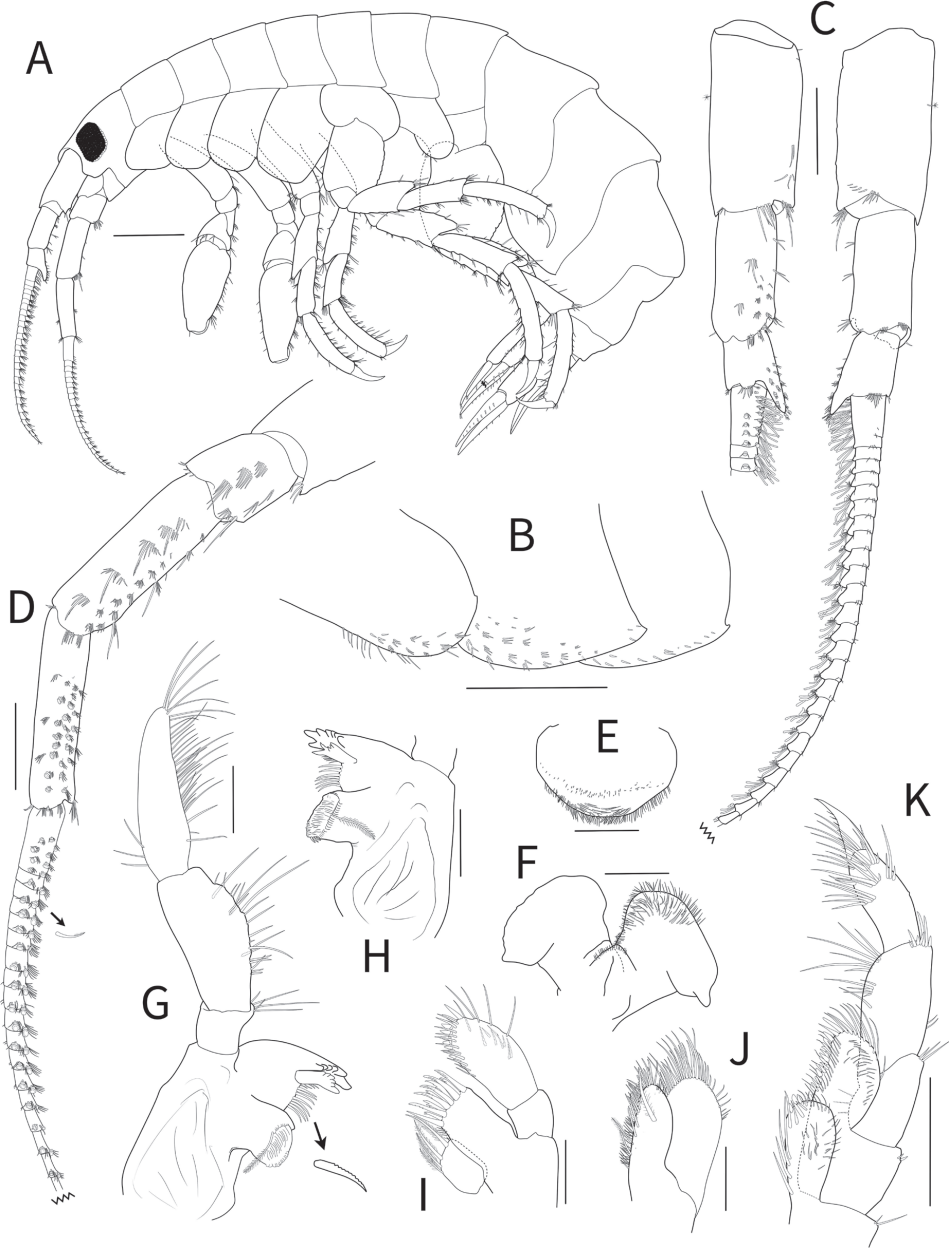
*Calliopiusulleungensis* sp. nov. holotype male, 13.6 mm **A** habitus **B** epimeron **C** antenna 1, medial margin and lateral margin **D** antenna 2, medial margin **E** upper lip **F** lower lip **G** left mandible **H** right mandible **I** maxilla 1 **J** maxilla 2. **K** maxilliped. Scale bars: 1.0 mm (**A, B**); 0.5 mm (**C, D, K**); 0.2 mm (**E–J**).

**Figure 4.**
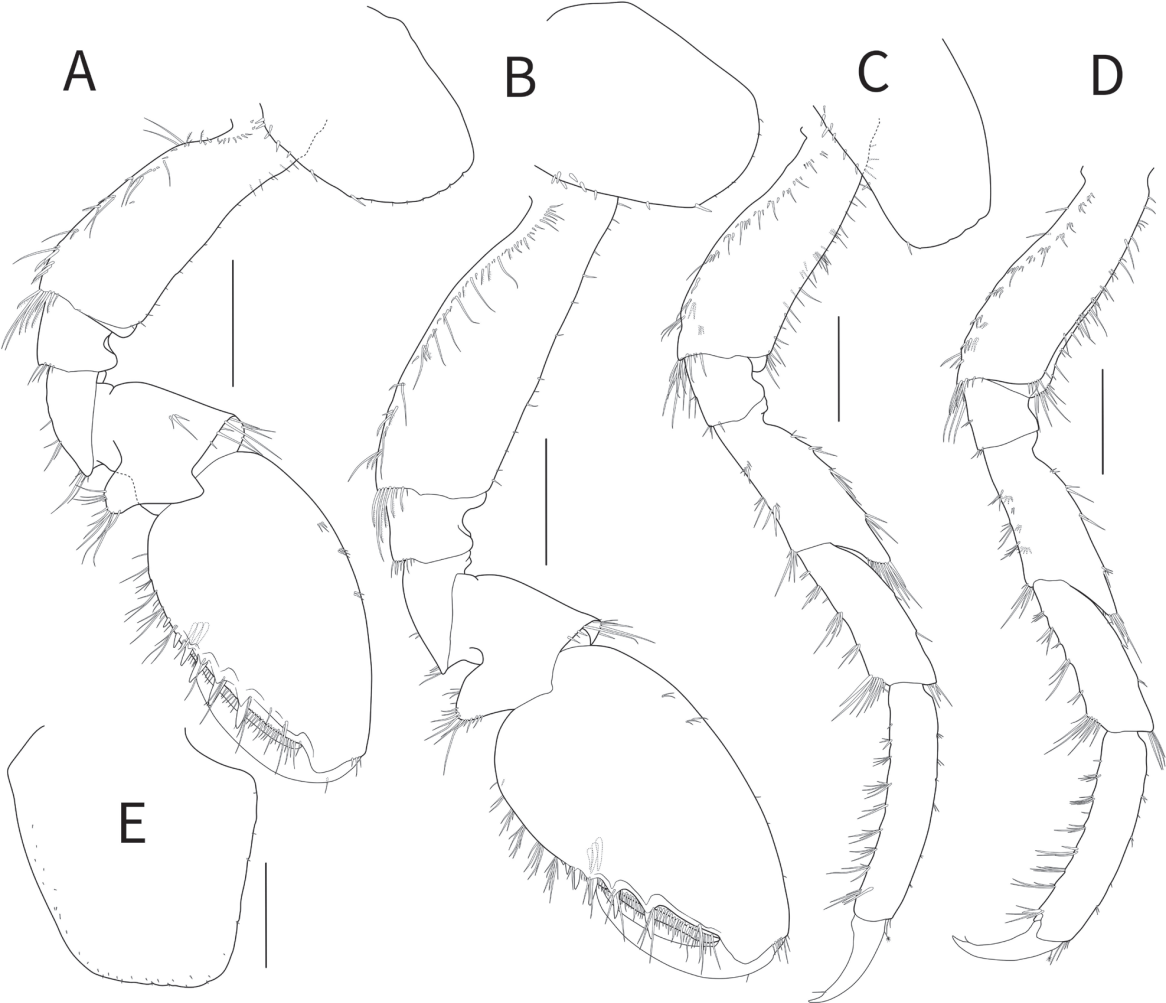
*Calliopiusulleungensis* sp. nov. holotype male, 13.6 mm **A** gnathopod 1 **B** gnathopod 2 **C** pereopod 3 **D** pereopod **E** coxa 4, left. Scale bars: 0.5 mm.

**Figure 5.**
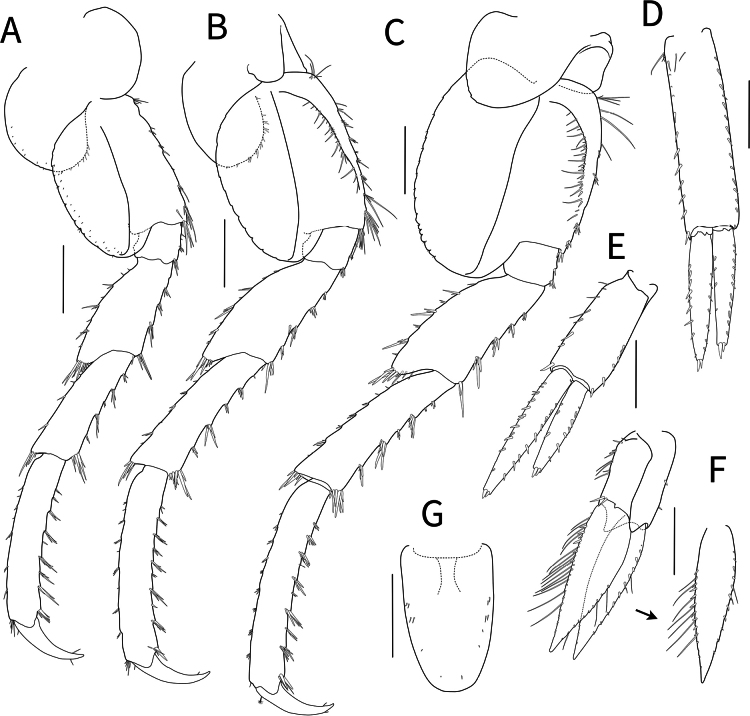
*Calliopiusulleungensis* sp. nov. holotype, male, 13.6 mm **A** pereopod 5 **B** pereopod 6 **C** pereopod 7 **D** uropod 1 **E** uropod 2 **F** uropod 3, outer ramus **G** telson. Scale bars: 0.5 mm.

**Figure 6.**
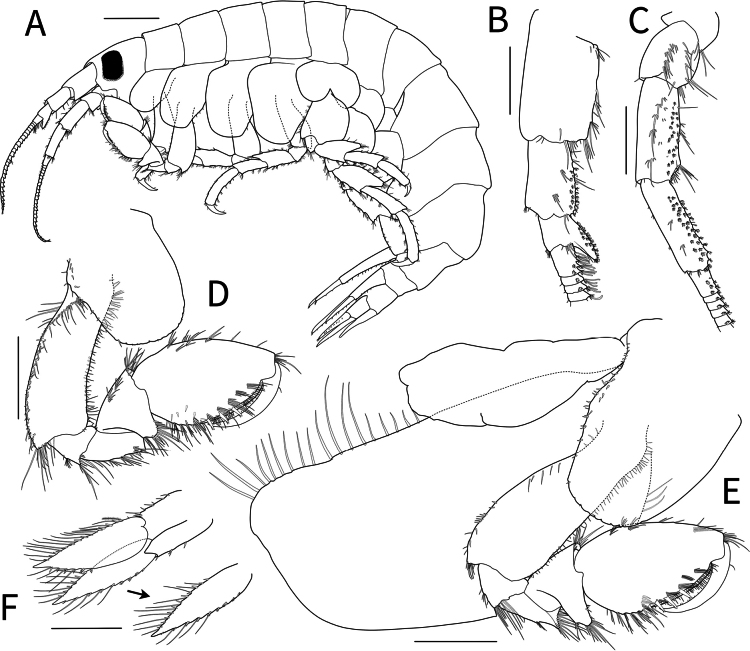
*Calliopiusulleungensis* sp. nov. paratype, female, 11.6 mm **A** habitus **B** antenna 1, medial margin **C** antenna 2, medial margin **D** gnathopod 1 **E** gnathopod 2 **F** uropod 3. Scale bars: 1.0 mm (**A**); 0.5 mm (**B–F**).

Figs 2 , 3 , 4 , 5 , 6 Korean name: Ul-leung- kal-li-o-pe-yeop-sae-u, new

##### Type material.

***Holotype***: • ♂, 13.6 mm, dissected (appendages on one slide), MABIK CR00257942, South Korea: Ulleungdo Island (site 1), Gyeongsangbuk-do, 37°30'30"N, 130°58'12"E, collected from floating algae (*Sargassumhorneri*) K.W. Kim leg., 24 May 2023. ***Paratypes***: • 1 ♀, 11.5 mm, dissected (appendages on one slide), DKUAMP202408; • 9 ♂♂, 28 ♀♀, DKUAMP202409, same station data as holotype.

##### Additional material.

• 2 ♂♂, 8 ♀♀ Ulleungdo Island (site 2), Gyeongsangbuk-do, 37°32'33"N, 130°50'28"E, collected by conical net, K.W. Kim leg., 23 May 2023; • 1 ♂, Taeha-ri, Seo-myeon, Ulleungdo Island, Gyeongsangbuk-do, 37°30'52"N, 130°47'36"E, collected by hand net, Y.H. Kim leg., 24 May 2023; • 5 ♂♂, 10 ♀♀ Bongpo-ri, Toseong-myeon, Goseong-gun, Gangwon-do, 38°14'32"N, 128°34'28"E, collected from brown algae, Y.H. Kim leg., 20 Jul 2023.

##### Diagnosis.

Eyes well developed, subrectangular. Antenna 1 calceolate, peduncular article 3 with distoventral process; flagellum callynophorate, longer than peduncle. Antenna 2 calceolate, densely setose, slightly flattened; gland cone bluntly pointed; flagellum longer than peduncle. Mandibles, incisor with six-seven dentate, lacinia mobilis on both sides, molar triturative. Gnathopods subchelate, moderate, subsimilar; propodus ovoid, palmar margin with numerous setae, six strong robust spines, palmar corner with four medial robust spines; dactylus falcate. Uropod 3 rami foliaceous. Telson linguiform, entire.

##### Description.

**Holotype, adult male**, MABIK CR00257942.

***Body*** (Figs 2A, 3A) 13.6 mm long, pleonite 7–urosomite 2 weekly carinated, laterally compressed; eyes well developed, subrectangular.

***Epimera*** (Fig. 3B), epimeron 1 posteroventral corner minutely pointed, with marginal simple setae, 10 clusters of setae ventrally; epimeron 2 posteroventral corner pointed, with 18 clusters of setae ventrally; epimeron 3 with posteroventral cusp, 12 ventral setae.

***Antenna 1*** (Fig. 3C) peduncular articles stout, cylindrical, less setose, articles 2–3 calceolate medioventrally, article 3 with obtuse short process distoventrally, length ratio of peduncular articles 1–3 = 1.00: 0.69: 0.35; flagellum longer than peduncle, 28-articulate, callynophore, two calceoli distomedially on each article from second flagellum; accessory flagellum minute.

***Antenna 2*** (Fig. 3D) setose; peduncular articles 2–3 short, peduncular articles 3–4 with medial setal rows; peduncular article 4, half of ventrodistal portion with calceoli; peduncular article 5 slender, with two or three rows of calceoli; length ratio of peduncular articles 3–5 = 1.00: 2.11: 2.20; flagellum slightly depressed, 23-articulate, two calceoli distomedially on each article from second flagellum.

***Upper lip*** (Fig. 3E) semicircular, apically round, pubescent.

***Lower lip*** (Fig. 3F), inner plate indistinct, densely pubescent; outer plate distally expanded, pubescent mediodistally; mandibular process developed.

***Left mandible*** (Fig. 3G), incisor with six blunt teeth, lacinia mobilis with six teeth; accessory setal row with eight setae between lacinia mobilis and molar; molar triturative surface well developed, with pappose seta; palp 3-articulate; article 1 short, distally setose; article 2 midmedially broadened, with unequal simple setae; article 3 narrowing distally, three apical setae; length ratio of articles 1–3 = 1.00: 3.51: 4.40.

***Right mandible*** (Fig. 3H) similar to left mandible; incisor with seven blunt teeth, lacinia mobilis with five apical teeth; accessory setal row of seven setae between lacinia mobilis and molar.

***Maxilla 1*** (Fig. 3I), inner plate subrectangular, with four pappose setae apically; outer plate, apical margin with 11 dentate setal teeth; palp biarticulate; article 1 short, unarmed; article 2 elongated ovate, swollen distally, with 11 robust setae.

***Maxilla 2*** (Fig. 3J), inner plate apex and medial margins setose with a plumose seta on surface; outer plate large, broad, with row of mediodistal setae.

***Maxilliped*** (Fig. 3K), inner plate subrectangular, medial margin with six plumose setae submarginally, apex with four plumose and four stout robust setae; outer plate semicircular, apex slightly beyond end of palp article 2, apex and medial margin straight with marginal blunt robust setae; palp 4-articulate, article 1 short, articles 2 with distal robust setae, article 3 distal half covered with rows of setae, article 4 falcate.

***Gnathopod 1*** (Fig. 4A) subchelate, densely setose; coxa with small robust setae posteriorly; basis subrectangular, relatively long, broadened distally, anterior margin with minute setae, posterolateral margins setose, with cluster of pinnate setae posterodistally; ischium short, 0.27× basis, with distal setae; merus subtriangular, 0.41× basis; carpus with subtriangular posterior lobe, with apical setae; propodus ovoid, longer than basis; palmar margin with numerous plumose setae, with six strong robust spines, palmar corner with four medial robust spines; dactylus elongated, falcate, 0.55× propodus.

***Gnathopod 2*** (Fig. 4B) subsimilar to gnathopod 1 but elongated; basis 1.1× that of gnathopod 1; propodus slightly longer; palmar corner with five medial robust spines.

***Pereopod 3*** (Fig. 4C) setose; basis subsimilar to that of gnathopod 1, anterior margin setose, merus with four cluster of robust setae, produced anterodistally with cluster of seta; carpus subrectangular, expanded distally, with postero-marginal cluster of thick setae; propodus rectangular, slightly convex; dactylus falcate, length ratio of basis–dactylus = 1.00: 0.22: 0.75: 0.65: 0.89: 0.37.

***Pereopod 4*** (Fig. 4D) similar to pereopod 3, slightly shortened. coxa (Fig. 4E) broad; length ratio of basis–dactylus = 1.00: 0.31: 0.77: 0.74: 0.89: 0.38.

***Pereopod 5*** (Fig. 5A), coxa bilobate, wider than long; basis subrectangular, expanded posteroventally with 10 minute setae, anterior margin with marginal short robust setae and distal long robust setae; ischium subquadrate; with antero-marginal robust setae; merus with robust setae on both margin, produced posterodistally, with cluster of seta; carpus subrectangular, expanded distally; propodus rectangular, slightly convex; dactylus falcate, length ratio of basis–dactylus = 1.00: 0.24: 0.77: 0.85: 1.06: 0.45.

***Pereopod 6*** (Fig. 5B) similar to pereopod 5, posterior lobe distally elongated, basis broader than that of pereopod 5, anterolateral margin with row of setae; length ratio of basis–dactylus = 1.00: 0.22: 0.79: 0.85: 1.06: 0.45.

***Pereopod 7*** (Fig. 5C) similar, but longer than either pereopods 5 or 6; basis broad, twice the area of that of pereopod 5, posterior lobe over end of ischium, anterodistal margin with short robust setae; length ratio of basis–dactylus = 1.00: 0.18: 0.70: 0.79: 1.01: 0.32.

***Uropod 1*** (Fig. 5D), peduncle subrectangular, each margin with dorsal row of robust setae.; outer ramus × 0.40 peduncle, with 11 robust setae on laterally, four robust setae on medially, two robust setae on apex; inner ramus × 0.5 peduncle, with nine dorsolateral robust setae and seven dorsomedial robust setae, apex on tow robust setae.

***Uropod 2*** (Fig. 5E), peduncle subrectangular; outer ramus 0.86× peduncle, with seven dorsolateral robust setae and five dorsomedial robust setae, apex on tow robust setae; inner ramus 1.45× peduncle, with 14 dorsolateral robust setae and nine dorsomedial robust setae, apex on tow robust setae.

***Uropod 3*** (Fig. 5F). peduncle short, both rami foliaceous; outer ramus 1.62× peduncle, with 11 dorsolateral and 14 dorsomedial robust setae; inner ramus subequal to outer ramus, with 21 dorsolateral and 9 dorsomedial robust setae.

***Telson*** (Fig. 5G) linguiform, entire, 1.61 times as long as wide, with 2 setules on each side.

**Paratype, adult ovigerous female**, DKUAMP202408.

***Body*** (Figs 2A, 6A) 11.6 mm long, laterally plump, coxae broader than male.

***Antenna 1*** (Fig. 6B) similar to that of male, peduncular articles shortened; length ratio of peduncular articles 1–3 = 1.00: 0.65: 0.32; flagellum 28-articulate.

***Antenna 2*** (Fig. 6C) similar to that of male, peduncular articles shortened; length ratio of peduncular articles 3–5 = 1.00: 1.58: 1.60; flagellum 25-articulate.

***Gnathopod 1*** (Fig. 6D) similar to that of male but reduced, more setose; propodus ovoid, longer than basis; palmar margin with numerous plumose setae, six strong robust spines, palmar corner with four medial robust spines.

***Gnathopod 2*** (Fig. 6E) subsimilar to gnathopod 1 but elongated; coxa expanded, similar in length with basis; basis 1.1× that of gnathopod 1; propodus slightly longer; palmar margin with numerous plumose setae, seven strong robust spines, palmar corner with four medial robust spines.

**Remarks.***Calliopiusulleungensis* sp. nov. resembles several Pacific region species, *C.behringi* Gurjanova, 1951 from King Island, Bering Sea, *C.pacificus* Bousfield & Hendrycks, 1997 from Alaska, USA, and *C.carinatus* Bousfield & Hendrycks, 1997 and *C.columbianus* Bousfield & Hendrycks, 1997 both from British Columbia, Canada, in having two to numerous rows of calceoli on the peduncular articles on the antennae. Among them, *C.behringi* and *C.carinatus* can be easily distinguished by their strong body carination, elongated posterodistal process on antenna 1 peduncular article 3.

In general, *Calliopiusulleungensis* sp. nov. is similar to *C.columbianus*. However, the new species can be distinguished from *C.columbianus* by the following characteristics (*C.columbianus* characters in parentheses): 1) eyes subrectangular (vs ovate); 2) antenna 1, posterodistal process of peduncular article 3 reaching half of flagellar article 1 (vs reaching the end of flagellar article 1); 3) antennae flagellum with fewer than 30 articles (vs with more than 30 articles, up to 40); and 4) maxilla 1, inner plate with 4 apical pappose setae (vs with 5 apical pappose setae). *Calliopiusulleungensis* is geographically close to *C.ezoensis*. The collection sites of *C.ezoensis* are on the southern and northeastern coasts of Hokkaido (Shimoji et al. 2020), connected to the collection site of *C.ulleungensis* across the Tsugaru Strait and La Pérouse Strait. The females of both species are morphologically similar, with two or more rows of calceoli and a short process on the third article of antenna 1 peduncle. However, the new species can be distinguished from *C.ezoensis* by the following characteristics (*C.ezoensis* characters in parentheses): 1) male antennae with two or more rows of calceoli (vs one row of calceoli); 2) male gnathopod 2 larger than gnathopod 1 (vs gnathopod 1 larger than gnathopod); 3) in female antenna 2 peduncular article 4–5, rows of calceoli start from 1/4 of the basal (vs from half of the basal).

##### Etymology.

The species name is derived from the type locality, Ulleungdo Island located off the East Sea of Korea.

##### Distribution.

Korea (Gangwon-do, Ulleungdo Island, East Sea).

### ﻿Key to species of the genus *Calliopius* from Western Pacific (modified from Bousfield and Hendrycks 1997)

**Table:** 

1	Pacific (Antennae, calceoli present in one or two rows on posterior surface of peduncular articles, especially in females)	**2**
–	Atlantic (Antennae, calceoli present in two to several rows on posterior surface of peduncular articles, especially in females)	**7**
2	Antenna 1, posterodistal process of peduncular article 3 elongate, extending along 4–6 basal flagellum; uropod 2, outer ramus short, 0.5× inner ramus	***Calliopiusbehringi* Gurjanova, 1951**
–	Antenna 1, posterodistal process of peduncular article 3 short, not extending or reaching end of flagellar article 1; uropod 2, outer ramus moderate, 0.7× inner ramus)	**3**
3	Pereonite 5 to pleonite 2 distinctly carinate, with dorsal tubercles; epimeron 2, facial setae in 5–7 submarginal rows	***C.carinatus* Bousfield & Hendrycks, 1997**
–	Pereonites to pleonites not or weakly carinate, without dorsal tubercles; epimeron 2, facial setae in two or three submarginal rows	**4**
4	Antennal flagella short (<20 articles); pereopods 5–7, dactyli large, heavy, > 1/3 length of propodus; maxilla 1, inner plate with 2 apical setae	***C.pacificus* Bousfield & Hendrycks, 1997**
–	Antennal flagella elongate (>20 articles); pereopods 5–7, dactyli small, < 1/3 length of propodus; maxilla 1, inner plate with four or five apical setae	**5**
5	Antenna 1, posterodistal process of peduncular article 3 reaching distal end of flagellar article 1; antennal flagella over 30 articles	***C.columbianus* Bousfield & Hendrycks, 1997**
–	Antenna 1, posterodistal process of peduncular article 3 not reaching end of flagellar article 1; antennal flagella usually 30 articles	**6**
6	Eye ovate; gnathopod 1 distinctly larger than 2 in male; uropod 3, outer ramus with seven laterally marginal robust setae	***C.ezoensis* Shimoji, Nakano & Tomikawa, 2020**
–	Eye subrectangular; gnathopod 1 slightly smaller than 2 in male; uropod 3, outer ramus with 11 laterally marginal robust setae	***C.ulleungensis* sp. nov.**
7	Uropod 3, rami conspicuously setose on inner and outer margins	**8**
–	Uropod 3, rami conspicuously setose on inner margin only	**9**
8	Antenna 1, posterodistal process of peduncular article 3 elongate, exceeding flagellar article 1; epimeron 2, facial spines in submarginal row	***C.laeviusculus* (Krøyer, 1838)**
–	Antenna 1, posterodistal process of peduncular article 3 short, length < flagellar article 1; epimeron 2, facial spines in three submarginal rows	***C.sablensis* Bousfield & Hendrycks, 1997**
9	Coxae 1–4, distal margin distinctly crenulate; pereopods 5–7, dactyli strong, length > 1/3 propodus	***C.crenulatus* Chevreux & Fage, 1925**
–	Coxae 1–4, distal margin nearly smooth; pereopods 5–7, dactyli short, slender, length < 1/3 propodus	***C.rathkii* (Zaddach, 1844)**

## Supplementary Material

XML Treatment for Calliopius

XML Treatment for Calliopius ulleungensis

## ﻿Additional information

### Author ORCIDs

Kyung-Won Kim https://orcid.org/0000-0003-2152-9555

Young-Hyo Kim https://orcid.org/0000-0002-7698-7919

### Data availability

All of the data that support the findings of this study are available in the main text.
